# Supplementary material for: Evaluating Influenza Vaccination Practices among COPD Patients
Source: Vaccines (Basel). 2023 Dec 22;12(1):14. doi: 10.3390/vaccines12010014 (PMC10820400; doi:10.3390/vaccines12010014)
Supplement: Supplementary file 1 [file vaccines-12-00014-s001.zip › Questionnaire.pdf]

Part one

<u>Socio-demographic and medical profile:</u>

- Age_____________
- Gender___________
- Educational level

  Not educated

  Primary

  Secondary

  High school

  Diploma

  Bachelor's degree

  Postgraduate studies

- Marital status

  Single

  In a relationship

- Income

  <1000 Jd/month

  ≥ 1000 Jd/month

- Duration of COPD_____________
- Have you been hospitalized for COPD in the last year?

  Yes
  No
  Don't know

- Are you a smoker?

  Yes
  Ex-smoker
  No

- Does anyone in your household smoke?

  Yes
  No
  I Don't know

Part two

CAT

I never cough							I cough all the time
I have no phlegm (mucus) on my chest at all							My chest is full of phlegm (mucus)
My chest does not feel tight at all							My chest feels very tight
When I walk up a hill or a flight of stairs I am not out of breath							When I walk up a hill or a flight of stairs I am completely out of breath
I am not limited to doing any activities at home							I am completely limited to doing all activities at home
I am confident leaving my home despite my lung condition							I am not confident leaving my home at all because of my lung condition
I sleep soundly							I do not sleep soundly because of my lung condition
I have lots of energy							I have no energy at all

Part three

Knowledge about COPD, influenza and influenza vaccine

	Yes	No	Don’t know
COPD is an infectious disease	○	○	○
Can genetics play a role in a person’s susceptibility to COPD?	○	○	○
Patients may experience flare-ups/exacerbations	○	○	○
Do you know the spirometry test?	○	○	○
Does the flu make the symptoms of COPD worse?	○	○	○
Do you know the benefits of using COPD inhalers?	○	○	○
Engaging in physical activities helps improve COPD symptoms.	○	○	○
Do you know how to correctly use COPD inhalers?	○	○	○
Does diet affect COPD symptoms?	○	○	○
Does your psychological state affect COPD symptoms?	○	○	○

- Influenza is the same as a common cold?

Yes
No
Don’t know

- Influenza is caused by bacteria.

Yes
No
Don’t know

- Influenza can spread from one person to another

Yes
No
Don’t know

- Antibiotics can be used to treat flu

Yes
No
Don’t know

- Is there a vaccine against flu?

Yes
No
Don’t know

- Does the vaccine have side effects?

Yes
No
Don’t know

- When is the appropriate time to take the flu vaccine?

(January-March)
(April-August)
(September- October)
(November- December)

Part four

Attitude towards Flu vaccine

	Strongly agree	Agree	Neutral	Disagree	Strongly disagree
I believe that I should receive the influenza vaccination	○	○	○	○	○
I believe that I get sick because of the influenza shot	○	○	○	○	○
My physician believes that I should receive the flu vaccine	○	○	○	○	○
Influenza vaccination prevents infection by the influenza virus	○	○	○	○	○

Part five

<u>Practice regarding COPD</u>

	Always	Most of the time	Sometimes	Rarely	Never
How often do you avoid exposure to smoking?	○	○	○	○	○
How often do you avoid exposure to dust/air pollution?	○	○	○	○	○
How closely do you follow a healthy diet?	○	○	○	○	○
How regularly do you engage in physical activities?	○	○	○	○	○

- In your opinion, how dangerous is the flu to your health ?
  (1 low to 5 high)

- How often did you get vaccinated against flu?

Once
More than once
Annually
Never

If previously vaccinated, the respondents will be directed to previous experience about flu vaccine otherwise, the participants will be directed to skip this section to the future vaccination intension question

- Did you feel any of the following side effects after receiving the vaccine? [you can choose more than one answer]

Fever
Headache
Nausea
Redness/soreness in the site of injection

Muscle aches
Fatigue
Other (Please specify) _____________________

- What was the severity of the side effects?

Severe
Moderate
Low

- Do you intend to get the flu vaccine this year?

Yes
No
Not sure

If the respondents answered no or not sure to the previous question, they will be directed to reasons behind vaccination hesitancy otherwise, the participants will be directed to submit the questionnaire

- What are the reasons for you not wanting to be vaccinated? [you can choose more than one answer]

  - I forgot it
  - I do not believe it is effective
  - I think it may be harmful
  - I got the flu although I had previously been vaccinated
  - The physicians do not recommend it
  - It is expensive
  - I don’t know its benefits

الجزء الاول

<u>المعلومات الاجتماعية والديموغرافية:</u>

- العمر_______________
- الجنس_________________
- المستوى التعليمي

غير متعلم
ابتدائي
ثانوي
مدرسة ثانوية
دبلوم
درجة البكالوريوس
الدراسات العليا

- الحالة الاجتماعية

أعزب
مرتبط

- الدخل الشهري

<1000 دينار شهريا
≤ 1000 دينار شهريا

- مدة الإصابة بالانسداد الرئوي الزمن___________
- هل دخلت المستشفى بسبب مرض الانسداد الرئوي المزمن في العام الماضي؟

نعم
لا
لا أعرف

- هل أنت مدخن؟

نعم
مدخن سابق
لا

- هل تتعرض للتدخين السلبي؟

نعم
لا
لا أعرف

الجزء الثاني

ما حالة مرض انسداد الشعب الهوائية المزمن (COPD) لديك؟ قم بإجراء اختبارCOPD Assessment Test™ (CAT)

سوف يساعدك هذا الاستبيان أنت وأخصائي الرعاية الصحية على قياس تأثير COPD (مرض انسداد الشعب الهوائية المزمن) على صحتك وحياتك اليومية. ويمكن لك وأخصائي الرعاية الصحية استخدام إجاباتك ودرجاتك في الاختبار للمساعدة في تحسين إدارة مرض الانسداد الرئوي المزمن والحصول على أكبر استفادة من العلاج.

.لطباعة الإستبيان رجاءً **اضغط هنا** اذا كنت تأمل في عمل الإستبيان بالكتابة على الورق

لكل عنصر موجود أدناه، ضع علامة (X) في المربع الذي يصف حالتك حالياً على أفضل نحو. تأكد من اختيارك لرد واحد فقط على كل سؤال.

مثال: أنا سعيد جدًا (0) (X) (2) (3) (4) (5) أنا حزين جدًا

								النتيجة
لا أكح مطلقاً	0	1	2	3	4	5	أعاني من الكحة طوال الوقت	
ليس عندي أي بلغم (مخاط) في صدري على الإطلاق	0	1	2	3	4	5	صدري ممتلئ عن آخره بالبلغم (المخاط)	
لا أشعر بضيق في صدري على الإطلاق	0	1	2	3	4	5	أشعر بضيق شديد في صدري	
لا تجدني أنهج عند أصعد تل أو عندما أصعد دور واحد من السلم	0	1	2	3	4	5	تجدني أنهج تماماً عند السير لأعلى التل أو عندما أصعد دور واحد من السلم	
لا يقتصر عملي في المنزل على القيام بأي أنشطة	0	1	2	3	4	5	قدراتي محدودة كثيرا عند القيام بالأنشطة بالمنزل	
أغادر منزلي في ثقة بالرغم من حالة رئتي	0	1	2	3	4	5	لا أشعر بالثقة مطلقًا في مغادرة منزلي بسبب حالة رئتي	
أنام بعمق	0	1	2	3	4	5	لا أنام بعمق بسبب حالة رئتي	
لدي الكثيرمن الطاقة	0	1	2	3	4	5	ليس لدي طاقة على الإطلاق	
							النتيجة الإجمالية	

الجزء الثالث

المعرفة حول مرض الانسداد الرؤي و الانفلونزا ومطعوم الانفلونزا

	نعم	لا	لا أعرف
الانسداد الرئوي المزمن مرض معد	○	○	○
الانسداد الرئوي المزمن مرض وراثي	○	○	○
قد يعاني مرضى الانسداد الرئوي المزمن أحياناً من تفاقم في الاعراض	○	○	○
الربو مرض مزمن	○	○	○
هل تعرف اختبار قياس كفاءة الرئة؟	○	○	○
هل تجعل الإنفلونزا أعراض انسداد الرئوي المزمن أسوأ؟	○	○	○
هل تعرف ما الفائدة من استخدام بخاخات الانسداد الرئوي المزمن؟	○	○	○
هل تعرف كيفية استخدام بخاخات الانسداد الرئوي المزمن بشكل صحيح؟	○	○	○
هل طبيعة الغذاء يؤثر على أعراض الانسداد الرئوي المزمن؟	○	○	○
هل الحالة النفسية تؤثر على أعراض الانسداد الرئوي المزمن؟	○	○	○

الإنفلونزا هي نفسها نزلات البرد؟

نعم
لا
لا أعرف

- تحدث الأنفلونزا بسبب البكتيريا.

نعم
لا
لا أعرف

- يمكن أن تنتقل الأنفلونزا من شخص إلى آخر

نعم
لا
لا أعرف

- يمكن استخدام المضادات الحيوية لعلاج الأنفلونزا

نعم
لا
لا أعرف

- هل هناك لقاح ضد الانفلونزا؟

نعم
لا
لا أعرف

- هل تعرف الفرق بين لقاحات الأنفلونزا الثلاثية والرباعية؟

نعم
لا
لا أعرف

- هل للقاح الانفلونزا آثار جانبية؟

نعم
لا
لا أعرف

- ما هو الوقت المناسب لأخذ لقاح الإنفلونزا؟

(يناير-مارس)
(سبتمبر - أكتوبر)
(نوفمبر - ديسمبر)

- ما هو عدد جرعات لقاح الإنفلونزا المطلوبة لإكمال دورة التطعيم الكاملة؟

جرعة واحدة- جرعتان
اكثر من جرعتين
ثلاث جرعات

الجزء الرابع

السلوك المتعلق بلقاح الانفلونزا

	أوافق بشدة	أوافق	محايد	لا أوافق	لا أوافق بشدة
أعتقد أنني يجب أن أتلقى لقاح الأنفلونزا	○	○	○	○	○
أعتقد أنني أمرض بسبب لقاح الأنفلونزا	○	○	○	○	○
يعتقد طبيبي أنه يجب أن أتلقى لقاح الأنفلونزا	○	○	○	○	○
التطعيم ضد الإنفلونزا يمنع العدوى بفيروس الانفلونزا	○	○	○	○	○

الجزء الخامس

الممارسة المتعلقة بالانسداد الرئوي

	دائما	معظم الوقت	أحيانا	نادرا	أبدا
ما مدى تتجنبك التعرض للتدخين؟	○	○	○	○	○
ما مدى تتجنبك التعرض للغبار/تلوث الهواء؟	○	○	○	○	○
ما مدى التزامك بالنشاطات الرياضية بشكل دوري؟	○	○	○	○	○
ما مدى اتبعاك لحمية غذائية صحية؟	○	○	○	○	○

الجزء السادس

الممارسات حول لقاح الانفلونزا

- كم مرة تلقيت لقاح الانفلونزا؟

مرة
أكثر من مرة
سنويا
أبدا

- في رأيك ما مدى خطورة مرض الانفلونزا على صحتك (1-5)

اذا كانت إجابة المستبين على السؤال السابق (ابدا) سينتقل تلقائيا الى السؤال المتعلق حول نيته لاخذ المطعوم مستقبلا

- ما هو نوع اللقاح الذي تلقيته؟

ثلاثي
رباعي

- هل شعرت بأي من الآثار الجانبية التالية بعد تلقي اللقاح؟ [يمكنك اختيار اكثر من اجابة]

حمى
صداع
غثيان
احمرار / ألم في مكان الحقن
آلام في العضلات
اجهاد
أخرى (ارجو النحديد) ____

- ما هي شدة الآثار الجانبية؟

شديدة
معتدلة
خفيفة

- هل تنوي الحصول على لقاح الإنفلونزا هذا العام؟

نعم
لا
لست متأكدًا

اذا كانت إجابة المستبين على السؤال السابق (نعم) سيتم تحويله تلقائيا الى انهاء الاستبيان اما اذا كانت اجابته غير ذلك سيتم توجيهه تلقائيا الى أسباب عدم اخذ المطعوم

- ما هي أسباب عدم التطعيم؟ [يمكنك اختيار اكثر من اجابة]

أنسى ذلك

لا أعتقد أنه فعال

أعتقد أنه قد يكون ضارا

أصاب بالأنفلونزا بعد اللقاح

الأطباء لا ينصحون به

أعتقد أنه مكلف
