# Supplementary material for: Evaluating Influenza Vaccination Practices among COPD Patients
Source: Vaccines (Basel). 2023 Dec 22;12(1):14. doi: 10.3390/vaccines12010014 (PMC10820400; doi:10.3390/vaccines12010014)
Supplement: Supplementary file 1 [file vaccines-12-00014-s001.zip › vaccines-2738089-supplementary.pdf]

Table S1. Factor analysis findings for communalities, loadings, and Cronbach's alpha on attitudes towards the influenza vaccine

Factor	Communalities	Loadings	Cronbach's Alpha
My physician believes that I should receive the influenza vaccine	0.578	0.760	0.71
Influenza vaccination prevents infection by the influenza virus	0.651	0.807	
I believe that I must receive the influenza vaccination	0.717	0.847	
I believe that I get sick because of the influenza shot	0.31	0.487	

Table S2. Factor analysis findings for communalities, loadings, and Cronbach's alpha on attitudes towards managing COPD

Factor	Communalities	Loadings	Cronbach's Alpha
1			0.72
How regularly do you engage in physical activities?	0.764	0.884	
How closely do you follow a healthy diet?	0.728	0.838	
2			0.85
How often do you avoid exposure to dust/air pollution?	0.884	0.954	
How often do you avoid smoking/exposure to smoking?	0.867	0.910	
